# Interprofessioal collaboration among Speech Language Pathologists and Nurses in Acute Care in Pakistan

**DOI:** 10.12669/pjms.37.2.3545

**Published:** 2021

**Authors:** Fariha Ambreen Ch, Muhammad Naveed Babur, Sajid Rashid, Maria Liaqat

**Affiliations:** 1 Fariha Ambreen Ch, M.Phil. (Rehab Sci) Visiting Faculty Member, ISRA University, Islamabad Campus, Islamabad, Pakistan; 2 Prof. Dr. Muhammad Naveed Babur, PhD (Rehab Sciences) Principal, ISRA Institute of Rehabilitation Sciences, ISRA University, Islamabad Campus, Islamabad, Pakistan; 3 Prof. Dr. Sajid Rashid Ch Multan Medical & Dental College, Multan, Pakistan; 4 Maria Liaqat, PP-DPT Assistant Professor, ISRA University, Islamabad Campus, Islamabad, Pakistan

**Keywords:** Coordination, Inter-professioal collaboration, Intensive care unit, Nurses and shared decision making, Speech language pathologists

## Abstract

**Objective:**

To find out inter-professional collaboration among speech-language pathologists and nurses in acute care in Pakistan.

**Methods:**

This was a cross sectional study which was conducted in all government and private hospitals of Islamabad and Rawalpindi having facility of ICUs after taking consent from authorities. The duration of study was six months from October 2018 to February 2019. A total number of 350 participants (200 nurses, 150 speech language pathologists) working in ICU of different private and government hospitals of Pakistan were included in the study. Standardized questionnaire of “: assessment of inter-professioal collaboration scale”: (AITCS) was circulated to nurses and speech language pathologists (SLPS) working in ICU with its subscale’s partnership, coordination, cooperation and shared decision making on a 5-point likert scale. Data was analyzed using SPSS version 21.Measure of mean was obtained by independent sample t-test. P- Value less than 0.05 was considered as significant.

**Results:**

Statistical analysis showed measures of mean differences obtained by t-test revealed significant differences at p<0.001 level between partnership scores of SLPS and nurses. This reveals good partnership between two disciplines. Measures of mean differences obtained by t-test revealed significant differences at p<0.001 level between partnership scores of SLPS and nurses. Both do not value each other in cooperation. Measures of mean differences obtained by t-test showed significant differences at p<0.001 level amongst coordination scores of SLPS and nurses. Both have good coordination. Measures of mean differences obtained by t-test revealed significant differences at p<0.001 level amongst shared decision-making scores of SLPS and nurses. Both are involved in shared decision making.

**Conclusion:**

Results show significant difference in partnership, coordination, and shared decision making. There is no significant difference in cooperation.

## INTRODUCTION

It is this partnership that creates an inter-professioal team designed to work on common goals to improve patient outcomes. “Collaborative interactions exhibit a blending of professional cultures and are achieved though sharing skills and knowledge to improve the quality of patient care”.^1^

WHO also stated in 2012 “Inter-professioal collaborative practices happen when multiple health care staffs from different professional trainings work together with patients, families, professions and communities to deliver the highest quality of care”.^2^

Speech therapists and the nurses have significant roles in acute settings of care. Nurses somehow spend more time with each patient, mainly in the ICU whereas speech language pathologists (SLPs) also play consultative part, giving checkups and recommendations. SLPs may be expert in establishing communication systems, they may not get appreciation of the broader communication problems facing patients in settings of acute care.^3^

The speech-language pathologist is responsible for the accurate assessment and intervention of people with problems like swallowing and communication difficulties, both nurses and speech language pathologists’ work along patients experienced stroke. It is evident that SLP and nurses coordinate during their clinical practice to make the patient outcomes better. Because of increasing difficulties of patient care, it is basic to build collaborations sooner in interdisciplinary training for purpose to increase quality of the care of patient. The collaboration amongst teams needs to be set as a standard for the training of the healthcare professionals, nurses and speech-language pathologists.^4^

The WHO also focuses SLPs to educate themselves and everyone, collaborate with various disciplines to understand and treat dysphasia and acknowledge the different impact of the disorder.^5^ The SLP takes into account a combination of expertise in clinic, knowledge, experience and evidence-based practice along with collaboration with other disciplines when working with patients of swallowing disorders.

According to the WHO, the collaboration amongst disciplines will result in improved coordination of health services appropriate referral to specialist resources, improved for patients with chronic diseases, and overall. “Collaborative practices can decrease patient’s length of stay in a facility, reduce patient’s medical complications, as well as reduce staff turnover, tensions and conflict among caregivers, most importantly, reduce mortality rates”.^1^

An acknowledged structure of literature shows that the social system of support is associated with more psychological wellness and to a decreased possibility of physical illness, it hence proved to be sure that the resources given by interpersonal relationships has a significant role to play in determining individuals adaptive functioning along with the health results, and to justify the basis regarding subject, two investigations can be used being the first one.^6^ The purpose of this study was to find out inter-professioal collaboration among speech-language pathologists and nurses in acute care in Pakistan as limited evidence is available in Pakistan on similar topic.

## METHODS

This was a Cross Sectional Study. The present study was conducted in all government and private hospitals of Islamabad and Rawalpindi having facility of ICUs after taking consent from authorities. The duration of study was six months from October 2018 to February 2019. Overall sample size Calculated was 377 through Rao soft with margin of error 5%, confidence interval 95% and population size 20000, 425 forms were distributed and 350 were received. Two hundred forms received from nurses and 150 from SLPs who were working in ICU. Non probability convenience sampling was used to collect data. SLPs and Nurses working in ICU of different Government and private sector hospitals were approached besides all other allied health care professionals working in ICU and all other health care professionals working on managerial positions.

This study was initiated after approval from advanced study & research committee (ASRC) of ISRA Institute of Rehabilitation Sciences, ISRA University Islamabad by IRB number F.1/IUIC-IIRS/ASRC-045/2018. Standardized questionnaire was used, The Assessment of Inter-professioal Team Collaboration Scale (AITCS), with its 48 items within four subscales: Partnership, Coordination, Cooperation, Shared Decision making (assessed on a 5-point Likert scale, administered among SLPs and Nurses practicing within acute settings, in Pakistan.

Standardized questionnaire of Assessment of inter-professioal collaboration scale (AITCS) was circulated among nurses and speech language pathologists (SLPs) working in ICU with its subscale’s partnership, coordination, cooperation and shared decision making on a 5-point likert scale. Data was analyzed using SPSS version 21. The Shapiro-Wilk test was applied to checked normality of data. Data was normally distributed**.** Measure of mean was obtained by independent sample t-test. Value less than 0.05 was considered significant.

## RESULTS

A total of 350 participants were included in this study in which male were 87(67 Nurses and 20 SLPs) and female were 363(132 Nurses and 131 SLPs). Participants age was between 24 to 49 years. The mean age was 32.2. Educational level showed 187 were master degree holder and 159 were bachelor degree holder. Total of 200 nurses and 150 SLPs participated in this study. Majority of them were having professional experience from three to 10 years in their relative fields.

The results of independent t-test among two groups is shown in Table-I. Independent sample t-test was used to analyze the data. There were four variables partnership, cooperation, coordination and shared decision making. In this table group wise comparison is shown of each variable. There are five columns which show domains of inter professional relationship, sample number, disciplines, mean, SD and the p-value. There is a significant difference in partnership, coordination and shared decision making. There is no significant difference in cooperation.

**Table-I:** Results of independent t- test among two groups.

*Serial no*	*Domains*	*No. of Participants*	*Disciplines*	*Mean*		*SD*	*P-value*
1	Partnership	350	SLP	56.87	SLP	6.10	0.000
			Nursing	60.33	Nursing	6.55	
2	Cooperation	350	SLP	61.51	SLP	6.99	0.083
			Nursing	62.46	Nursing	2.78	
3	Coordination	350	SLP	30.97	SLP	2.12	0.000
			Nursing	33.51	Nursing	2.21	
4	Shared decision making	350	SLP	50.98	SLP	5.95	0.001
			Nursing	52.62	Nursing	3.49	
5	Total score	350	SLP	200.38	SLP	17.941	0.000
			Nursing	208.52	Nursing	14.31	

The score of questionnaires was measured from 0 to 100 where 0 is bad and 100 is good. The mean score of most domains are above 50. Mean score of partnership (SLP= 56.87, Nursing= 60.33). Measures of mean differences obtained by t-test revealed significant differences at p<0.001 level between partnership scores of SLPs and Nurses. This reveals good partnership between two disciplines. The mean score of cooperation (SLP= 61.51, Nursing= 62.46). Measures of mean differences obtained by t-test revealed non-significant differences at p<0.001 level between cooperation scores of SLPs and Nurses. Both do not value each other in cooperation. The mean score of coordination (SLP= 30.97, Nursing= 33.51). Measures of mean differences obtained by t-test revealed significant differences at p<0.001 level between coordination scores of SLPs and Nurses. Both have good coordination. The mean score of decision making (SLP= 50.98, Nursing= 52.62). Measures of mean differences obtained by t-test revealed significant differences at p<0.001 level between shared decision-making scores of SLPs and Nurses. Both are involved in shared decision making.

## DISCUSSION

Nursing is mainly dependent on partnership. Basic purpose was to know about basic characteristics of partnerships in health care systems and to form strategies to improve health care.^7^ Knowing the importance and benefits of the framework it is still found to be difficult to act on it and work according to the framework given. This study suggests and forces the system to incorporate rules and frameworks to guide professionals on how to act and be better at providing health care services.^8^

This study shows how important inter professional collaboration is achieving the desired outcomes. Practice done with collaboration demands stamina, energy and strength. Different professionals working together need to respect each other’s space and working capacities of each other. The problem-based learning curriculum is permeating nursing schools. Students need to be taught the importance of inter professional collaboration to prepare them for future. Outcomes of choice can be achieved through collaborative. There is synergism whenever there is a new partnership is formed which acts as a standard of inter professional relationship. This association must be acknowledged so that there is job satisfaction, better patient outcomes.^9^-^11^

Several features of effective primary health care teams and the related knowledge and skills that professionals require as effective team members are identified. “Effective teamwork requires specific cognitive, technical, and affective competence”.^12^

Current study talks about the inter-professional collaborative working experiences of professionals working along different families on two aspects of increased social requirement. The findings propose that, although the way that professionals make the concept their practice may provide resistant attempts to collaborate effectively, a complicated interplay of various factors involved. Finally, incorporating this work with the findings of other different studies, an explanatory model is established.^13^

The intensive care unit (ICU) is known to be an active, multifaceted and, sometimes, very stressful working environment that includes ongoing exposure to the current complexities of inter-professioal team operating. Lack of communication is considered as examples of poor collaboration amongst different health care professionals. Better communication between different health professionals lead to better outcomes of the patients. This study shows how better and improved communication leads to easier achieving to the common goals and outcomes and how it makes the professionals recognize the role of their colleagues.^14^

The aim of all the knowledge about all the components of health care and awareness about roles is to improve interventions of health care procedures and outcomes. Summary includes how patient is affected by poor and better collaborative skills of professionals working together for same purpose towards same goals.^15^

Most Shared decision making (SDM) models are unsuccessful to evaluate the importance of an inter-professional style. Those that considered at least two professionals met only a few of the elements of inter-professioal collaboration and had limited and defined description of SDM processes. Although models were considered as logically adequate, only half were tested and few were made using an explicit process.^16^

Shared decision making (SDM) frameworks in healthcare have been limited to the patient and the physician duo. Initial step towards promoting an inter-professional approach towards SDM in health care, current study shows how an inter-professional and interdisciplinary group formed and gained harmony on a new inter-professional SDM framework.^17^

A study conducted in Canada for inter-professional collaborative decision making among primary health care practitioners showed that inter-professional collaboration leads to shared decision making. In our study shared decision has significant impact in collaborative interdisciplinary process among SLPS and nurses working in ICU.^18^

Shared decision making is important to informed consent of patient and patient centered care. Till now SDM do not support the patient doctor duo, even though care is given by these teams working together for improved patient outcome. Collaboration is necessary in-home care, where patient grows the most. This literature will show if it is possible to practice SDM in IP home care.^19^ A study was conducted in which more than 50% of participants showed positive attitudes towards physician’s key role in team, effective communication among team members and role of team work in assuring patients’ satisfaction. Shared decision making is known as a way by which a healthcare decision is made by professionals working along the patient. A lot of diagnostic and therapeutic procedures in primary care combine more than one type of health expert.^20^

When it comes to the inter professional reachability about shared decision-making the working team sits together puts the patient treatment and benefit at first takes in account the patient preferences and enables the patient to have more control over the treatment.^21^ When care is provided by team work or through collaborative process, there is convincing evidence to prove better response of the patients. There is a lot of research available that proves that when practice is patient centered, the level of satisfaction improves.

### Limitations of the study

The study is specific to some hospitals of Islamabad and Rawalpindi only which can be done in other hospitals as well (private sector, NGOs etc.) to increase the generalizability of the research. The study is cross-sectional which can either be done with a longitudinal study design (collect data at several point of time).

## CONCLUSION

Intensive care unit is known to be dynamic, complex and usually very stressful working environment that includes exposure to complexity of inter-professioal team operating in it. Results show significant difference in partnership, coordination, and shared decision making. There is no significant difference in cooperation. This study reflected positive attitude towards interdisciplinary collaboration between SLPS and Nurses.

### Author`s Contribution:

**FA:** Conceived, designed.

**MNB:** Manuscript writing, final editing and he is also Responsible and accountable for the accuracy or integrity of the work.

**SR**: Analysis, data interpretation.

**ML** Statistical analysis, editing manuscript.
